# Supplementary material for: Fano resonance assisting plasmonic circular dichroism from nanorice heterodimers for extrinsic chirality
Source: Sci Rep. 2015 Nov 5;5:16069. doi: 10.1038/srep16069 (PMC4633605; doi:10.1038/srep16069)
Supplement: Supplementary Information [file srep16069-s1.doc]

**Supplemental Information**

**Fano resonance assisting plasmonic circular dichroism from nanorice heterodimers for extrinsic chirality**

Li Hu^1,3^, Yingzhou Huang^1^,*, Liang Fang^1^, Guo Chen^1^, Hua Wei^1^, Yurui Fang^2^,*

^1^ Soft Matter and Interdisciplinary Research Center, College of Physics, Chongqing University, Chongqing, 400044, P. R. China

^2^ Department of Applied Physics, Chalmers University of Technology, Göteborg, SE 412 96, Sweden

^3^ School of Computer Science and Information Engineering, Chongqing Technology and Business University, Chongqing, 400067, China

Corresponding Authors. Emails: [yzhuang@cqu.edu.cn](mailto:yzhuang@cqu.edu.cn) (Y. Huang), [yurui.fang@chalmers.se](mailto:yurui.fang@chalmers.se) (Y. Fang)

**The influence of materials on plasmon** **resonance.** Considering the two nanorices in each dimer were of the same size here, the materials of the two nanorices should be different. Therefore, various groups of nanorice materials were investigated. Figure S1 shows the extinction spectra of the heterodimer (l=240 nm, d=60 nm, g=10 nm, n=1.1) with different materials illuminated by LCP and RCP light respectively. As figure S1(b) shown, when the heterodimer consists of the same material (Au-Au), the two extinction spectra are identical which excited by LCP and RCP respectively. While the heterodimers consist of Au and other materials，there are abviously different of the extinction cross section excited by LCP and RCP, as figure S1(b-e) shown.

**
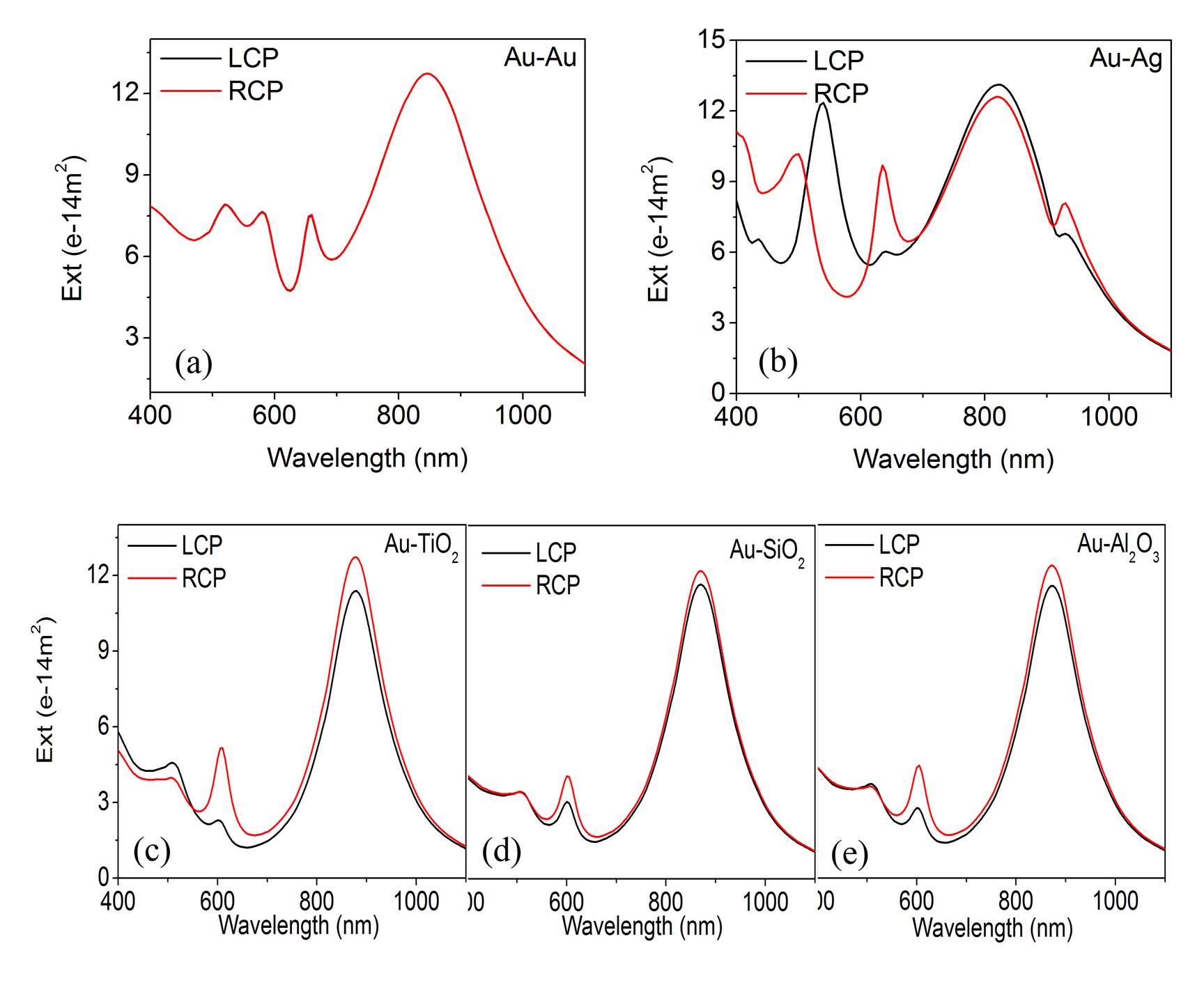
**

**Figure S1 |** **The influence of materials on plasmon resonance.** Extinction spectra of the heterodimer with different materials (*l=240 nm, d=60 nm, g=10 nm,θ=π/4*) excited by CPL (black: LCP, red: RCP). (a) Au-Au; (b) Au-Ag; (c) Au-TiO_2_; (d) Au-SiO_2_; (e) Au-Al_2_O_3_. CPL: circularly polarized light.

**The effect of the structure factors on plasmon resonance.** The structure factors (e.g. size and shape) play a great impact on the surface plasmon and Fano resonance. Figure S2(a) show the extinction cross section of Au and Ag nanorice with various sizes but the same aspect ratio (*l*/*d*= 4) excited by linear polarized light. As Figure S2(a) shown, the intensity of the resonant modes especially the quadruple mode increases markedly with the size of Ag and Au nanorice increasing, and the resonant wavelength have a significant red-shift . At the same time, the distance increases between the dipole and quadruple modes. Figure S2(b) shows the extinction spectra of the Au-Ag heterodimer excited by circularly polarized light (CPL). Fig S2(c) shows the extinction cross section of Au and Ag nanorice with various aspect ratio (l/d = 3, 4, 6) excited by linear polarized light. It can be seen the variation trend of Au nanorice and Ag nanorice is similar, and the Au nanorice change smaller. Figure S2(d) shows the extinction spectra of the heterodimer excited by LCP and RCP light .


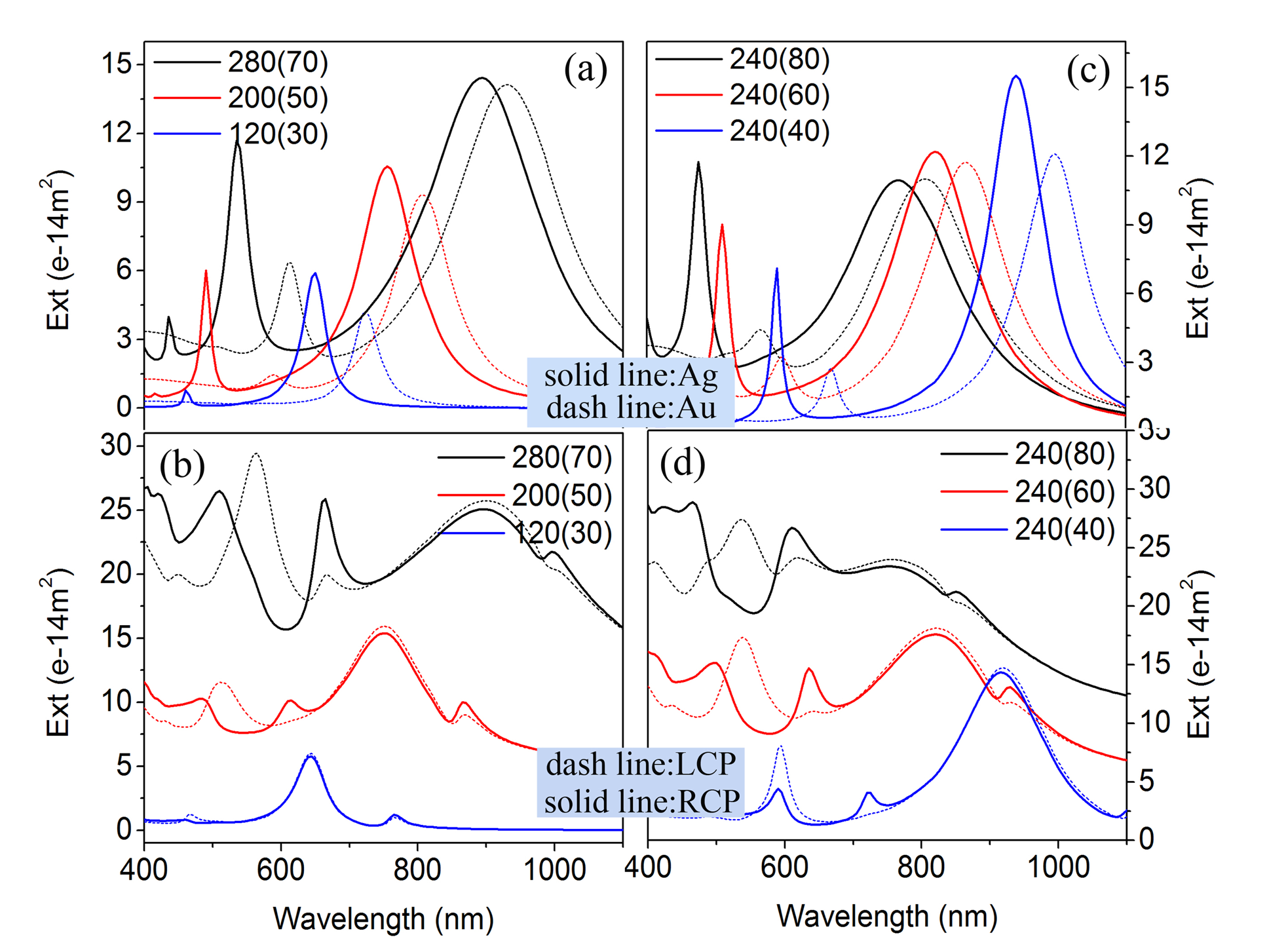


**Figure S2** **| The effect of the structure factors on plasmon resonance..** (a) The extinction spectra of individual Ag nanorice and Au nanorice excited by linear polarized light (*l/d=4, g=10 nm*). (b) The extinction spectra of Au-Ag heterodimer excited by LCP and RCP light (*l/d=4, g=10 nm*). (c) The extinction spectra of single Ag nanorice and Au nanorice excited by linear polarized light (*l/d=3,4,6,* *g=10 nm*). (d) The extinction spectra of Au-Ag heterodimer excited by LCP and RCP light. Set a heterodimer as (*l*(*d*)) in figure.

**Sensitivity of the Fano resonances to the surroundings.** Fig S3 (a) shows the extinction spectra of Au-Ag heterodimer in different surroundings excited by linearly polarized light. We can see that there are two obvious peak relatively. Fig S3 (b) shows the linear plot of resonance peak shifts of different order modes as a function of refractive index n. The *FOM* of mode 1 from 6.6 to 10.1, the *FOM* from 4.2 to 4.3 of the mode 2. Fig S4 (a) and (c) show the extinction spectra of Au-Ag heterodimer in different surroundings excited by LCP and RCP. Fig S4 (b) and (d) show the linear plot of resonance peak shifts of different order modes as a function of refractive index n. The *FOM* of mode L1 from 5.8 to 6.2 and mode R1 from 15.6-16.1, the *FOM* from 4.8 to 5.0 for the mode L2 and R2.


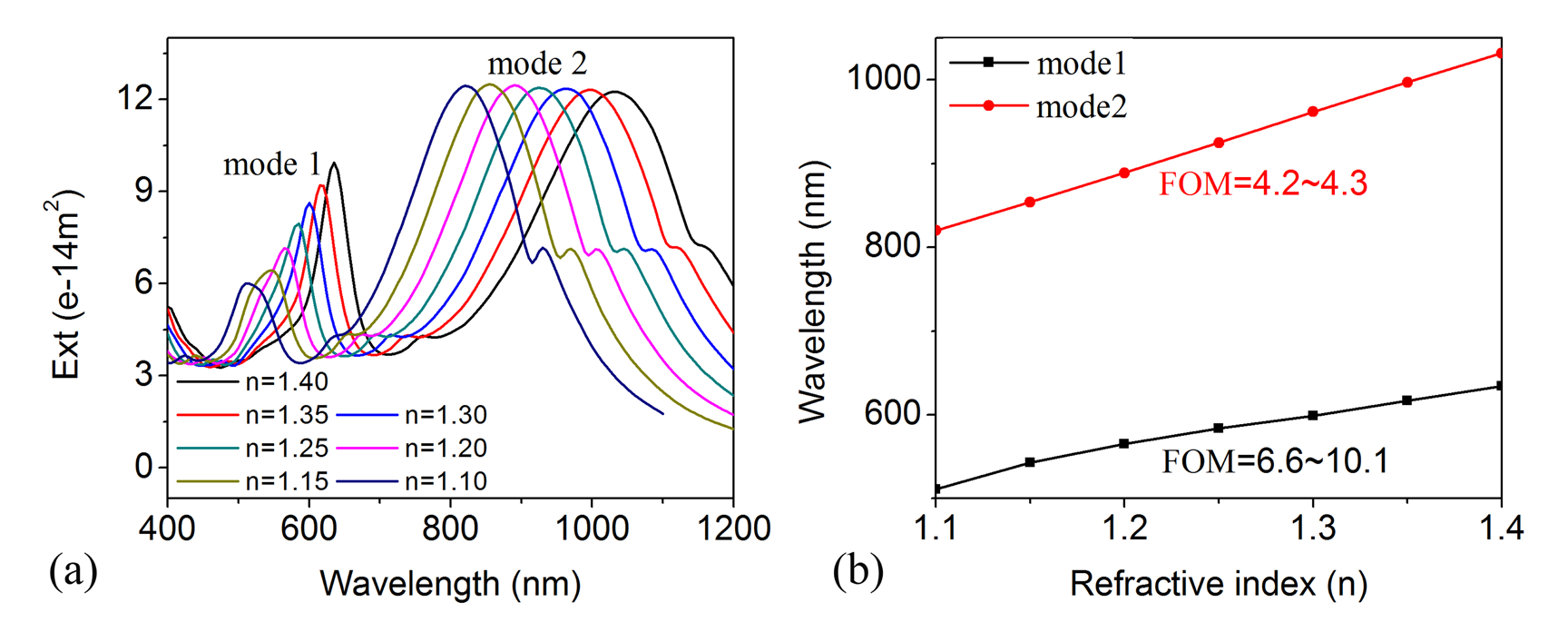


**Figure S3 | Sensitivity of the Fano** **resonances to the surroundings.** (a) Extinction spectra of Au-Ag heterodimer ($l=240nm,d=60nm,g=10nm$) in different surroundings excited by linearly polarized light. (b) Linear plot of resonance peak shifts of different order modes as a function of refractive index n. *FOM* of each mode is calculated.


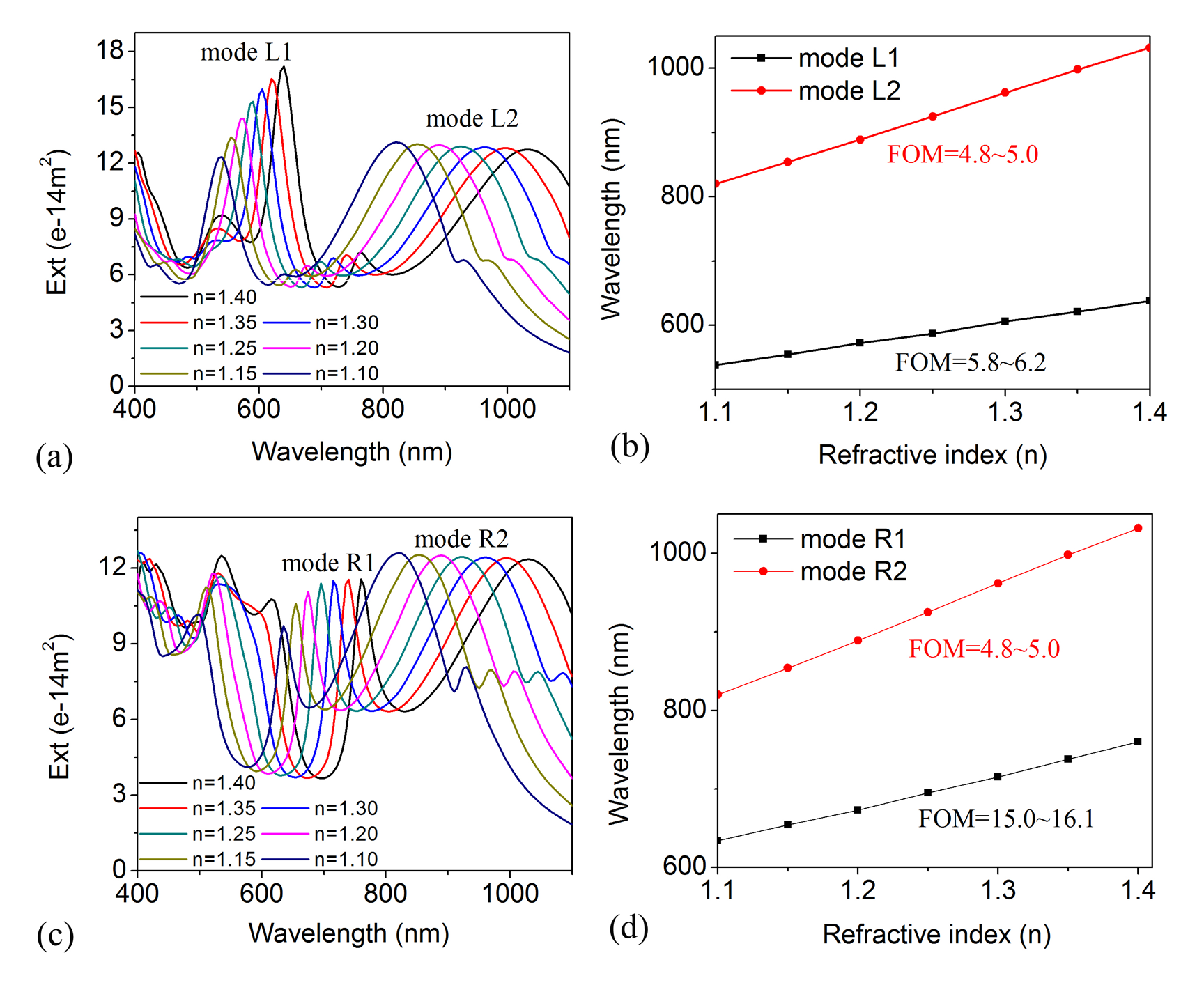


**Figure S4 | Sensitivity of the Fano resonances to the surroundings.** (a, c) Extinction spectra of Au-Ag heterodimer ($l=240nm,d=60nm,g=10nm$) in different surroundings excited by LCP and RCP. (b, d) Linear plot of resonance peak shifts of different order modes as a function of refractive index n (LCP and RCP, respectively). *FOM* of each mode is calculated.
